# Isolation, Characterization, and Degradation Performance of the 17β-Estradiol-Degrading Bacterium *Novosphingobium* sp. E2S

**DOI:** 10.3390/ijerph14020115

**Published:** 2017-01-25

**Authors:** Shunyao Li, Juan Liu, Minxia Sun, Wanting Ling, Xuezhu Zhu

**Affiliations:** Institute of Organic Contaminant Control and Soil Remediation, College of Resources and Environmental Sciences, Nanjing Agricultural University, Nanjing 210095, China; 2015103034@njau.edu.cn (S.L.); liujuan@njau.edu.cn (J.L.); 2013103044@njau.edu.cn (M.S.)

**Keywords:** 17β-estradiol, estrogen, degradation, *Novosphingobium* sp., cow manure

## Abstract

A 17β-estradiol (E2)-degrading bacterium E2S was isolated from the activated sludge in a sewage treatment plant (STP). The morphology, biological characteristics, and 16S ribosomal RNA (rRNA) gene sequence of strain E2S indicated that it belonged to the genus *Novosphingobium*. The optimal degrading conditions were 30 °C and pH 7.0. The ideal inoculum volume was 5% (*v/v*), and a 20-mL degradation system was sufficient to support the removal ability of strain E2S. The addition of extra NaCl to the system did not benefit the E2 degradation in batch culture by this strain. Strain E2S exhibited high degradation efficiency with initial substrate concentrations of 10–50 mg·L^−1^. For example, in mineral salt medium containing 50 mg·L^−1^ of E2, the degradation efficiency was 63.29% after seven days. In cow manure samples supplemented with 50 mg·L^−1^ of E2, strain E2S exhibited 66.40% degradation efficiency after seven days. The finding of the E2-degrading strain E2S provided a promising method for removing E2 from livestock manure in order to reduce the potential environmental risks of E2.

## 1. Introduction

Estrogens, which are important endocrine-disrupting compounds (EDCs), can be divided into two major groups: natural estrogens, such as estrone (E1), 17β-estradiol (E2), and estriol (E3), and synthetic estrogens, such as ethinyloestradiol (EE2) and diethylstilbestrol (DES) [1]. Like many other natural estrogens, E2 can affect aquatic fauna, even at extremely low concentrations [2,3]. Male fish suffer from feminization when exposed to ambient aquatic E2 at concentrations of 1–10 ng·L^−1^ [4]; even in the presence of E2 concentrations of 0.1–1 ng·L^−1^, reproductive disorders have been observed [5]. Moreover, E2 can accumulate through the food chain and can cause adverse health effects in humans [4]. E2 contributes to sexual disruption due to its high estrogenic activity and its recalcitrance to decomposition in the environment. As a result, it has attracted increasing public attention in recent decades [6,7].

Human and livestock excretions are the major sources of natural estrogens [8]. Liu et al. [9] investigated two adult males, 133 premenopausal women, and 30 pregnant women, and found that they excreted 1.5, 4.71, and 347 μg·day^−1^ of E2 per person via urine, respectively. Human excretion is often treated in municipal wastewater treatment plants (WWTPs), and WWTP effluent contains very low concentrations of E2 with inactive polar conjugates; however, bacterial enzymes in WWTPs can still convert E2 into its active form. Thus, estrogenic activity is frequently detected in the terminal effluent and it can cause negative effects [3,10,11]. To address this issue, efficient methods of removing E2 from effluent have been employed to prevent the negative effects of E2 pollution in aquatic biota. In contrast, the removal of E2 from livestock manure is presently a worldwide concern.

Livestock manure can act as a larger source of E2 than human excretion due to the lower standards for livestock manure disposal into the environment. Liu et al. [12] reported the sources of estrogens in Shanghai, China, including WWTPs, wastewater discharge from livestock farms, untreated or simply digested sewage from rural households, and run-off from farmland with livestock manure applied and irrigated with livestock wastewater. They found that 56.8 g·day^−1^ of estrogens (in E2 equivalents) were discharged by livestock, nearly twice that excreted by humans (35.2 g·day^−1^). Similar research in the United States revealed that 10–30 kg·day^−1^ of E2 was produced by dairy and swine [13], which was far higher than the amount excreted by humans. These studies show that E2 in livestock manure is a global concern that must be addressed.

Numerous approaches have been assessed for their ability to decrease estrogenic activity and residual E2 concentrations in the environment, including photocatalytic degradation, adsorption techniques, and biodegradation or biotransformation [14]. For example, Whidbey et al. [15] attempted to eliminate E2 via photocatalysis; however, the residual photodegradation products still retained estrogenic activity in water. Patrick et al. [16] assessed the efficiency of E2 phototransformation, and achieved a quantum yield of 0.06 under irradiation at 254 nm in an aqueous solution; they argued that photodegradation was an effective chemical technology for reducing E2 pollution. Several studies on adsorption technology found that negligible amounts of estrogens were removed [17,18,19]. Fukuhara et al. [20] tested the efficacy of activated carbon (AC) in adsorbing E2, and achieved an equilibrium E2 concentration of less than 1 mg·L^−1^ in pure water with initial E2 concentrations of 1.3–67.6 mg·L^−1^. Despite these physiochemical techniques, more cost-effective and environmentally friendly techniques are still wanted to address E2 pollution.

Biodegradation is an optimal option for removing E2 pollution. Waste composting is widely used to treat livestock manure. Zheng et al. [21] observed 80% degradation efficiency for both E1 and E2 after composting dairy cow waste for three months. Suzuki et al. [22] combined livestock waste composting with methane fermentation to remove E2 from digestion liquid, and were able to achieve terminal E2 concentrations of 0.002~0.011 μg·L^−1^. These investigations inspired our search for functional strains that could directly degrade E2. Isolating functional strains that can mineralize estrogens directly may be useful in increasing the degradation efficiency of natural estrogens. Some estrogen-degrading strains have been reported in the last few decades [23,24,25,26,27]. However, only a few documented E2-degrading bacteria have been tested for their ability to remove E2 from cow manure, and isolation of more strains with high E2-degrading ability is still needed.

The aim of this study was to isolate the E2-degrading strain from the estrogen-polluted environment, and to evaluate the degradation efficiency of E2 by the functional strain in batch culture and cow manure. As previously reported [14,28], activated sludge was a most probable source material for estrogen-degrading bacteria. An E2-degrading strain E2S was isolated from the activated sludge that was collected from a domestic sewage treatment plant (STP). Experiments were then performed to test the E2-degradation capacity and environmental adaptability of strain E2S in batch culture, as well as its E2-degradation efficiency in cow manure. The results of this study not only enrich the pool of E2-degrading genes, but also provide a basis for using E2-degrading strain in realistic livestock manures to remove E2, and contribute to efforts to decrease the environmental risks of E2.

## 2. Materials and Methods

### 2.1. Chemicals and Growth Media

E2 (C_18_H_24_O_2_; >98% purity) was purchased from Sigma-Aldrich (St. Louis, MO, USA). Its molecular weight, solubility in water, octanol-water partition coefficient, and vapor pressure were 272.38 g·mol^−1^, 5.4–13.3 mg·L^−1^, log *K*_ow_ 3.8–4.0, and 3 × 10^−8^ kPa, respectively. High-performance liquid chromatography (HPLC)-grade pure methanol and acetonitrile were purchased from TEDIA (Fairfield, OH, USA). A highly concentrated stock solution of E2 (1000 mg·L^−1^) was prepared in methanol.

The mineral salt medium (MSM) (pH 7.0 ± 0.2) [29] was composed of 1.50 g (NH_4_)_2_SO_4_, 0.50 g KH_2_PO_4_, 1.91 g K_2_HPO_4_·3H_2_O, 0.5 g NaCl, 0.20 g MgSO_4_·7H_2_O, and 1 mL trace element solution in 1 L ultra-pure water (UP, 18.25 MΩ·cm). The trace element solution contained 0.015 g·L^−1^ CuSO_4_·5H_2_O, 0.10 g·L^−1^ CoCl_2_·6H_2_O, 0.05 g·L^−1^ ZnCl_2_, 0.01 g·L^−1^ NiCl_2_·6H_2_O, 0.425 g·L^−1^ MnCl_2_·4H_2_O, and 0.01 g·L^−1^ Na_2_MoO_4_·2H_2_O. The estrogen mineral salt medium (EMM, pH 7.0 ± 0.2) was composed of MSM supplemented with E2 at the test concentrations. To create the liquid EMM, E2 was transferred with the designated concentrations into sterile flasks, the methanol was allowed to volatilize, and then the MSM was added. For the solid EMM plates, E2 was mixed with the melting MSM, the medium was poured into plates, and a stream of sterile air was used to volatilize the methanol. The Luria–Bertani medium (LB, pH 7.0 ± 0.2) [30] contained 10.0 g tryptone, 5.0 g yeast extract, and 10.0 g NaCl in 1 L of UP water. The Mueller–Hinton medium (M-H, pH 7.3 ± 0.1) [31] was composed of 300.0 g of beef dehydrated infusion, 17.5 g casein hydrolysate, and 1.5 g starch in 1 L of UP water. Solid medium plates were prepared by adding 18 g·L^−1^ of agar to the liquid media.

### 2.2. Enrichment and Isolation of E2-Degrading Strains

For the enrichment procedure, 100 mg·L^−1^ E2 was added into 100 mL of liquid EMM in a 250-mL Erlenmeyer flask before inoculating with 5% (*w/v*) of activated sludge sample, and the mixture was cultured in a reciprocal shaker at 30 °C and 150 r·min^−1^. Five percent (*v/v*) of this mixture was transferred into fresh liquid EMM containing 100 mg·L^−1^ E2 every seven days, and this process was repeated four times. Residual E2 concentrations in the enrichment solutions were measured by HPLC with ultraviolet light (UV) detection (Shimadzu LC-20AT, Tokyo, Japan).

Next, 1 mL of the enrichment culture that exhibited obvious E2 removal was transferred into sterilized tubes containing sterile water to prepare serial 10-fold dilutions. One hundred microliters of samples diluted by factors of 10^−2^ to 10^−6^ were plated on separate EMM plates containing 100 mg·L^−1^ of E2 and cultivated at 30 °C for seven days. Morphologically distinct colonies were selected for purification by repeated streaking until a single colony was obtained.

Then, the possible single colony underwent secondary screening. Each purified single colony was amplified by culturing it in liquid LB medium in a rotary shaker at 30 °C and 150 r·min^−1^ for 24 h to prepare the cell suspension. The cell suspension was centrifuged at 8000 r·min^−1^ for 4 min, and the pellet containing bacteria was then collected, washed twice, and re-suspended in sterile MSM, which was adjusted to an optical density at 600 nm of 1.0.

The degradation system comprised 5% (*v/v*) cell suspension in 20 mL of EMM containing 100 mg·L^−1^ of E2 in 50-mL Erlenmeyer flasks. The mixture was cultivated in the rotary shaker at 30 °C and 150 r·min^−1^ for seven days. Residual E2 concentrations were detected with HPLC/UV. All experiments were performed in triplicate.

### 2.3. Identification of Strain E2S

The cell morphology of strain E2S was observed by a Hitachi transmission electron microscope system (×8000, Zoom-1 HC-1 80 kV). The physiological and biochemical characteristics were determined according to the bacterial identification manual [32]. Gram reaction, glucose fermentation test, starch hydrolysis test, gelatin hydrolysis test, nitrate reductase, phenylalanine deaminase, citrate utilization, methyl red test, Voges-Prokauer test, indol test, H_2_S production, and catalase test were carried out. For the 16S rRNA gene sequence analysis, the genomic DNA of strain E2S was extracted by using a DNA Extraction Kit (Tiangen Biotech, Beijing, China). Universal bacterial primers [33] 27 forward (F) (5′-AGAGTTTGATCCTGGCTCAG-3′) and 1492 reverse (R) (5′-TACCTTGTTACGACTT-3′) were used to amplify the 16S rRNA gene via PCR under the following conditions: 94 °C for 4 min, 35 cycles of 60 s at 94 °C, 60 s at 53 °C, 100 s at 72 °C, and a terminal step at 72 °C for 10 min, followed by 4 °C for 10 min [34]. The amplification products were sequenced by Nanjing GenScript Biotechnology Company (Nanjing, China) [29]. The partial nucleotide 16S rRNA gene sequence of E2S was compared with sequences in the EzTaxon Database (http://www.ezbiocloud.net/taxonomy). The neighbor-joining method was processed to construct a phylogenetic tree based on evolutionary distances in MEGA 6.0 (Tempe, AZ, USA), and an analysis of 1000 bootstrap sets was performed to evaluate the tree topology.

### 2.4. Minimum Inhibitory Concentrations of Strain E2S

The minimum inhibitory concentrations (MICs) of four antibiotics in strain E2S were investigated using the agar dilution method, which was standardized by the Clinical and Laboratory Standards Institute (CLSI) [31]. Erythromycin (EM), oxytetracycline (OTC), tetracycline (TC), and penicillin (P) as four representative veterinary antibiotics at concentrations of 0.25–128 µg·mL^−1^ were experimented [35]. Strain E2S was inoculated on M-H agar plates and incubated at 30 °C for 48 h, and the cell growth situation was recognized.

### 2.5. Degradation of E2 by Strain E2S in Culture Solution

To investigate the degradation dynamics of E2 and the growth curve of strain E2S, the degradation efficiency of E2 (as the percentage of the initial concentration) and corresponding cell counts were assessed on a daily basis for seven days. Using timed sampling and the dilution counting method, the degradation efficiency of E2 and cell counts were calculated, respectively. The operational conditions were the same as those used in the secondary screening process.

The effects of different environmental conditions on E2 degradation by strain E2S were experimented, including temperature (20–42 °C), pH (5.0–9.0), inoculum volume (0%–15% (*v/v*)), system volume (10–50 mL), NaCl concentration (0–25 g·L^−1^), and initial E2 concentration (10–100 mg·L^−1^). Non-inoculation treatments were set as black control (CK). All experiments were performed in triplicate.

### 2.6. Removal of E2 in Cow Manure by E2S

Cow manure samples were collected from a dairy farm in Nanjing, China. Each fresh cow dung sample was collected randomly from five sites within a 25-m^2^ area, mixed in a plastic cask, and transported to the lab immediately for the E2 removal analysis.

Because cow manure contains low concentrations of E2 (µg·kg^-1^) [36], 50 mg·kg^−1^ of E2 was added to the cow manure samples to simulate high E2 pollution. Then, 10.0 g of fresh freeze-dried E2-supplemented cow manure was mixed with 10.0 mL of liquid MSM to keep the moisture content at 50%. The mixture was placed on a 9 cm × 9 cm sterilized plate, evenly inoculated with 5% (v/w) E2S cell suspension, and then cultured at 30 °C for seven days. The cow manure samples were over-turned every 12 h. Residual E2 concentrations were detected with HPLC/UV on day 3, 5, and 7. All experiments were performed in triplicate.

### 2.7. E2 Concentration Analysis in Aqueous Solutions and Cow Manure

The residual E2 in the MSM solution was detected with HPLC/UV (LC-20AT; Shimadzu, Japan) following the method described by Xu et al. [37]. Briefly, 20 mL of methanol was added into flasks to dissolve residual E2, and the mixture was ultrasonicated for 30 min and then filtered through 0.22-µm polytetrafluoroethylene filters. The following settings were used for the HPLC/UV detection: Intertsil ODS-SP-C_18_ column (Shimadzu, Tokyo, Japan) (5 μm, 150 mm × 4.6 mm); a mobile phase of acetonitrile and water (70/30, *v/v*) at a flow rate of 1 mL·min^−1^; UV detection of E2 at 280 nm; and a 20-μL injection volume.

E2 was detected in cow manure following the method reported by Fu et al. [36]. Briefly, 15 mL of ethyl acetate was added to 1-g samples, the residual E2 was extracted by ultrasonication (Shanghaiziyi, Shanghai, China) for 1 h, and the mixture was centrifuged (Luxiangyi, Shanghai, China) at 4500 r·min^−1^ for 30 min. The supernatant was collected, concentrated with nitrogen, dissolved in methanol, and diluted to 50 mL in UP water. Then, 5 mL of sample was run through a C_18_ solid-phase extraction column activated with 5 mL of methanol and 5 mL of UP water. The compounds adsorbed on the C_18_ column were eluted with methanol and ethyl acetate (1/1, *v/v*), and the elution was collected and concentrated with nitrogen. The final target compounds were dissolved in 2 mL of methanol and filtered through a 0.22-μm organic filter before detection with HPLC/UV using the same parameters as described above.

The degradation efficiency (%) of E2 in batch culture or cow manure was calculated as follows:

(1) $$\mathrm{Degradation}\;\mathrm{efficiency}\;(\%)=\frac{C_{\mathrm{Initial}}-C_{\mathrm{Residual}}}{C_{\mathrm{Initial}}}\times 100\%$$

where *C*_Initial_ and *C*_Residual_ represented the initial and the final concentrations of E2 in culture solution or cow manure, respectively.

### 2.8. Statistical Analysis

All figures and data processing were performed in Microsoft Office Excel 2010 (Microsoft, Inc., Redmond, WA, USA). Variance was analyzed in SPSS ver. 13.0 (SPSS, Inc., Chicago, IL, USA), and *p* < 0.05 was considered to indicate significance. The data are expressed as the means and standard deviation of three parallel tests, represented by the error bars in the figures.

## 3. Results

### 3.1. Isolation and Identification of the E2-Degrading Bacterium E2S

**Figure 1:**
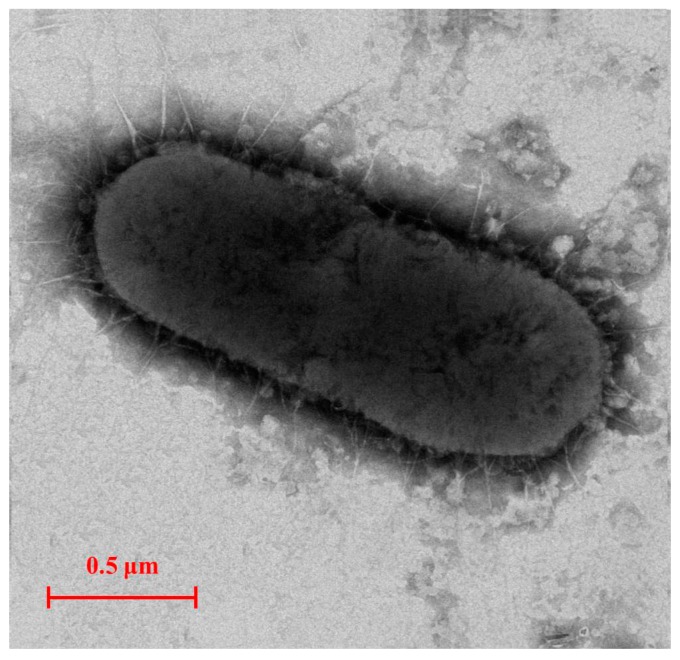
Transmission electron micrograph image of strain E2S (×8000, Zoom-1 HC-1, 80 kV).

An E2-degradating bacterium E2S was isolated from the activated sludge of a STP in Nanjing, China. E2S is rod shaped with a size of 0.5 μm × 1.8 μm and lacks a flagellum (Figure 1). The analysis of the physiological and biochemical characteristics of E2S show that it is gram negative; it lacks the ability to produce indol or H_2_S, hydrolyze starch, use sodium citrate, decompose glucose to produce pyruvate, or hydrolyze gelatin. By contrast, the glucose fermentation, nitrate reductase, phenylalanine dehydrogenase, methyl red, and catalase tests were positive.

**Figure 2:**
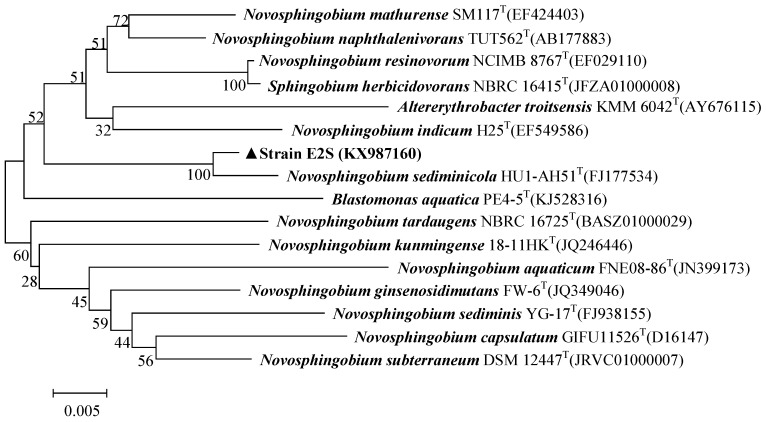
Phylogenetic tree of *Novosphingobium* sp. strain E2S and closely related species. The tree was constructed using the neighbor-joining method based on 16S rRNA gene sequences. The bar represents a genetic distance of 0.005.

The 16S rRNA gene sequence of E2S was submitted to GenBank (accession number, KX987160). A BLAST analysis of the 16S rRNA gene sequence from strain E2S against sequences in the EzTaxon Database revealed that strain E2S was >98% identical to strains of *Novosphingobium* sp. Figure 2 shows a phylogenetic tree of strain E2S and related species. The initial characterization of E2S supports that it is a strain of *Novosphingobium* based on its morphology, physiological, and biochemical characteristics, and 16S rRNA gene sequence.

### 3.2. Antibiotics Resistance of Strain E2S

Antibiotics resistance is another important physiological characteristic of a certain bacterium. The MICs of four common antibiotics (EM, OTC, TC, and P) in strain E2S was determined (Table 1). It was resistant to EM, OTC, TC, and P below concentrations of 4, 16, 64, and 0.5 µg·mL^−1^, respectively, and the MICs of EM, OTC, TC, and P in strain E2S was 8, 32, 128, and 1 µg·mL^−1^, respectively. According to the interpretation standards issued by CLSI, strain E2S is tetracycline resistant (MIC ≥ 16 µg·mL^−1^), penicillin susceptible (MIC ≤ 8 µg·mL^−1^), and moderately sensitive to erythromycin (0.5 µg·mL^−1^ ≤ MIC ≤ 8 µg·mL^−1^).

### 3.3. Degradation of E2 by Strain E2S in Batch Culture

**Figure 3:**
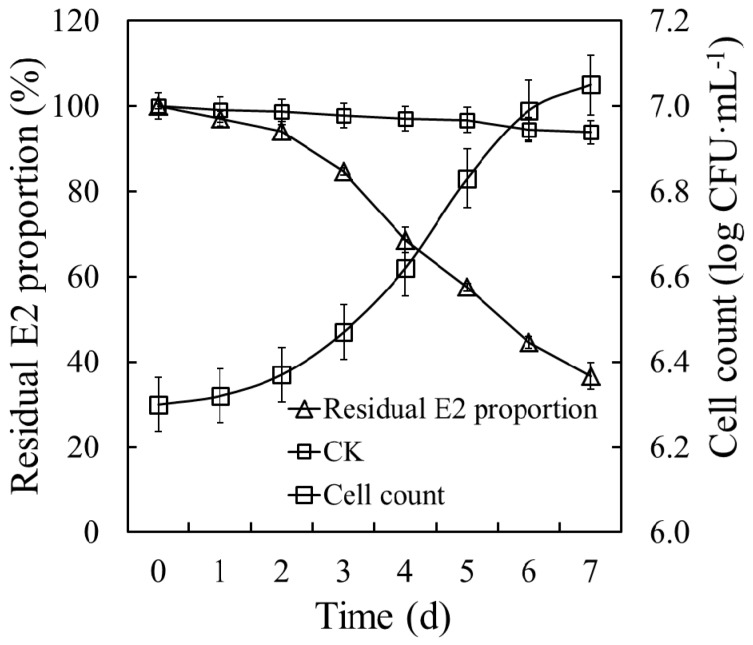
E2 degradation dynamics and growth curve of strain E2S in estrogen mineral salt medium (EMM). CK represents the non-inoculation treatment; CFU means colony forming unit.

The degradation curve and growth trend of E2S were assessed (Figure 3). Strain E2S exhibited a significant E2 degradation efficiency of 50 mg·L^−1^, and 63.29% of E2 was removed within seven days. The E2 degradation kinetics was represented by the formula *C* = 1.35E+02e^-1.50E-01t^, where *C* represents E2 residual concentration, and t represents incubation time, with *R*^2^ = 0.9306 and a half-life of 4.6 days. Cell count increased significantly during the degradation period, from 6.30 to 7.05 log CFU·mL^−1^. By contrast, the small variations in E2 concentration in the CK revealed noticeable levels of routine wastage of E2. Based on the decrease in the residual E2 concentrations and increase in biomass reflected by the cell counts, strain E2S was able to use E2 as its sole carbon source.

**Figure 4:**
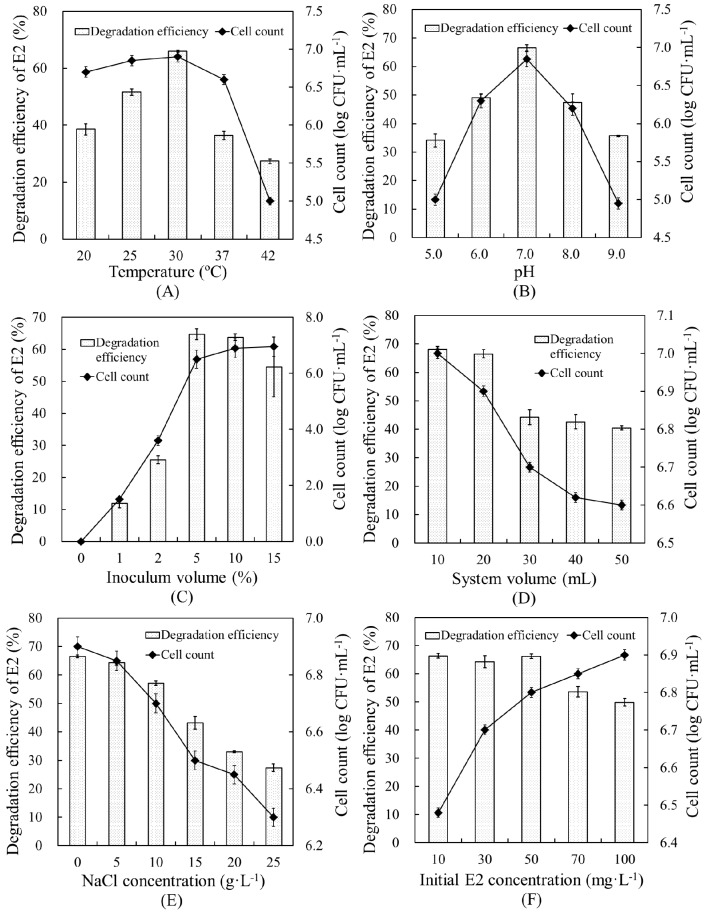
Effects of environmental parameters on E2 removal in EMM by strain E2S: (**A**) temperature, (**B**) pH; (**C**) inoculum volume; (**D**) system volume; (**E**) NaCl concentration; and (**F**) initial E2 concentration. The error bars represent standard deviations.

The effects of various environmental conditions on the E2 degradation efficiency and cell growth of strain E2S were evaluated, and the optimal conditions of six environmental factors for promoting degradation were determined (Figure 4). A temperature of 30 °C and pH of 7.0 were the most suitable conditions for promoting E2 removal by strain E2S, yielding degradation efficiencies of 65.94% and 66.46%, respectively. In addition, at the highest degradation efficiencies, cell count was correlated with degradation efficiency. This verified the close relationship between degradation capacity and bacterial biomass.

Inoculum volume and reaction system volume ranges of 0%–15% and 10–50 mL were optimized, respectively. An inoculation volume of 5% of the reaction system yielded the highest degradation efficiency, 64.7%, showing that greater bacterial biomass resulted in higher degradation efficiency. Increasing the total volume of the system did not promote E2 removal, but resulted in lower cell counts, which fell from 7.0 to 6.6 log CFU·mL^−1^. This revealed that strain E2S was likely an aerobic bacterium. Therefore, we determined 20 mL to be the ideal system volume, which yielded a degradation efficiency of 66.50%.

Increasing the NaCl concentration did not benefit the degradation efficiency. Higher NaCl concentrations resulted in lower degradation efficiency and cell counts. Therefore, there was no need to add NaCl to the reaction. Finally, regarding the initial E2 concentration, we determined that 10–50 mg·L^−1^ was optimal for system performance, as it had little effect on strain E2S growth, but performed well.

Overall, cell count was positively correlated with the degradation efficiency of E2. This study confirmed the importance of optimizing environmental factors to promote the E2 degradation efficiency of strain E2S.

### 3.4. Removal of E2 in Cow Manure by Strain E2S

**Figure 5:**
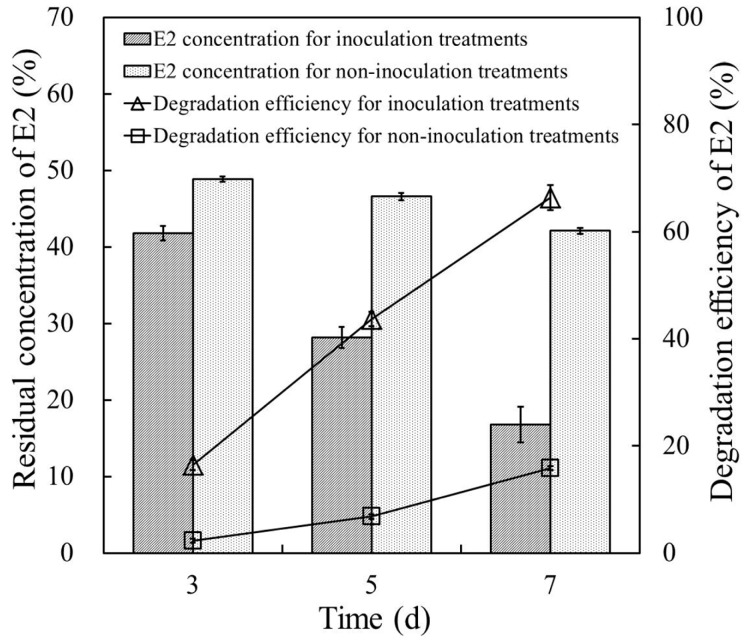
Removal of E2 in cow manure samples with and without inoculation of strain E2S. The error bars represent standard deviations.

Figure 5 shows the E2 degradation efficiency of strain E2S in cow manure supplemented with 50 mg·L^−1^ E2. Residual concentrations of E2 in cow manure for E2S-inoculation treatments were 41.8, 28.2, and 16.8 mg·kg^−1^ after cultivation for three, five, and seven days, respectively, which were significantly much higher than those for non-inoculation controls (CK; 48.9, 46.6, and 42.1 mg·kg^−1^). The E2 degradation efficiency in cow manure with strain E2S increased from 16.45% to 66.40% when cultivation time extended from three to seven days; that is, the degradation efficiency of E2 increased by ~50 percentage points over the final four days of incubation. By contrast, the degradation efficiency of E2 for non-inoculation controls was only 15.8% after seven days. These results indicated that strain E2S participated in removing E2 from the cow manure; this should be explored in greater detail in future research.

## 4. Discussion

A number of reports of functional natural and synthetic estrogen-degrading bacteria have been published in recent years. Table 2 includes a partial list of estrogen-degrading bacteria that have been isolated from activated sludge, lakes, sandy soil, and sediment since 2007, including their degradation efficiencies for each target compound. Functional strains of estrogen-degrading bacteria typically belong to the genera *Pseudomonas*, *Bacillus*, *Acinetobacter*, *Rhodococcus*, and *Agromyces*, among others, and have the capacity to degrade E1, E3, nonylphenol (NP), DES, and EE2. Different strains exhibit varying degradation efficiencies, even within genera. For example, strains *Bacillus* sp. E2Y1 and E2Y4 require four and six days, respectively, to remove the same amount of target contaminant.

As the universal estrogenic compound, E2 has been extensively researched, and most strains listed in Table 2 exhibit roughly equal capacity to degrade E2. In this study, we isolated a *Novosphingobium* sp. with the ability to degrade E2, which we named strain E2S. The genus *Novosphingobium* is famous for its excellent degradation efficiency of chlorinated compounds, mono- and polycyclic aromatic compounds, and other compounds, with notable species including *Novosphingobium aromaticivorans* and *Novosphingobium pentaromativorans* US6-1^T^ [38]. However, publications on estrogen degradation by *Novosphingobium* are scarce compared to those on polycyclic aromatic hydrocarbon (PAH) degradation, as research on estrogen degradation has emerged more recently. Only a little information is available for selected bacteria such as *Novosphingobium tardaugens* ARI-1 and *Novosphingobium* sp. JEM-1. The discovery of E2S highlights the importance of this functional genus and provides convincing proof of the outstanding ability of *Novosphingobium* to treat xenobiotics.

In this research, strain E2S was enriched in an initial E2 concentration of 100 mg·L^−1^. This applied high selection pressure compared with previously reported programs. The optimal substrate concentrations (10–50 mg·L^−1^) for E2 removal illustrate that E2S is well adapted to high E2 concentrations and that E2S is not exhausted under high E2 conditions. Moreover, it maintains its high degradation efficiency, offering further advantages.

Using a novel approach, we investigated the ability of strain E2S to remove residual E2 in livestock manure by inoculating it in manure supplemented with 50 mg·kg^−1^ of E2. As expected, low levels of E2, amounting to only 16.8 mg·kg^−1^, remained after seven days of treatment, equivalent to a degradation efficiency of 66.4%, thereby confirming that strain E2S was able to remove E2 from cow manure. However, the concentration of E2 in the manure (50 mg·kg^−1^) was much higher than environmentally relevant concentrations, which are often in the μg·kg^−1^ range. Therefore, future research should assess whether strain E2S can perform effectively under realistic situations and degrade environmentally relevant concentrations of E2.

Harsh environmental conditions prevent microbial growth and reduce degradation efficiency. Given the sensitively of strain E2S to various environmental conditions, future research should investigate the methods of enhancing its environmental adaptability. For example, immobilization technologies can protect cells from environmental stressors and minimize competition with native microorganisms [43,44,45]. Our laboratory has performed research using the immobilized strain ARI-1 to remove estrogen from water and cow manure; however, the feasibility of immobilizing strain E2S remains to be determined.

Strain E2S should be assessed for its broader capacity to degrade estrogens. Based on the results of other studies (Table 2), most strains with the ability to degrade E2 can also remove E1, and even exhibit strong broad-spectrum effects on E3 and EE2, including *Acinetobacter* sp. BP8 and BP10 and *Pseudomonas aeruginosa* BP3 and BP7. In addition, *Stenotrophomonas maltophilia* ZL1 can transform E2 into E1 and then further convert E1 into the amino acid tyrosine for use in protein biosynthesis. Compared with other strains also belonging to the same genus, *Novosphingobium* ARI-1 and *Novosphingobium* sp. JEM-1, strain E2S displays a distinct degradation ability of E2. ARI-1 holds the degradation ability of E1, E2, and E3, JEM-1 can degrade E1, E2, and EE2, while strain E2S is proven to degrade E2 in this work. Whether E1 and E3, which have lower estrogenic activities than E2, can be degraded by E2S remains to be determined in future research.

Although this study provides only preliminary results, there is no doubt that strain E2S promoted the elimination of residual E2 in livestock manure, providing a possible method for treating E2 contamination and reducing the potential risks associated with E2 pollution from livestock manure.

## 5. Conclusions

An E2-degrading bacterium, strain E2S, was isolated from the activated sludge in a sewage treatment plant. This strain belonged to the genus *Novosphingobium*. It exhibited obvious E2 degradation capability in culture solution with the optimum environmental conditions of 30 °C and pH 7.0. E2 removal from cow manure was achieved by inoculation with strain E2S, suggesting that it has high potential for practical applications. The E2-degrading bacterium strain E2S not only enriched the pool of E2-degrading genes, but also provided a basis for using the E2-degrading strain in realistic livestock manures to remove E2. The E2-degrading bacterium has demonstrated its capacity to contribute to efforts to decrease the potential environmental and public health risks of E2, and as such, should be a subject for future studies.

## Figures and Tables

**Table 1:** Minimal inhibitory concentrations (MICs) of four antibiotics in strain E2S.

Antibiotics	Concentrations (µg·mL^−1^)									
	128	64	32	16	8	4	2	1	0.5	0.25
Erythromycin	−	−	−	−	−	+	+	+	+	+
Oxytetracycline	−	−	−	+	+	+	+	+	+	+
Tetracycline	−	+	+	+	+	+	+	+	+	+
Penicillin	−	−	−	−	−	−	−	−	+	+

**Table 2:** Partial list of estrogen-degrading bacteria isolated since 2007.

Bacteria	Estrogens	Degradation Efficiency	Reaction Time	Initial Concentrations (mg·L^−1^)	Reference
*Agromyces* sp. LHJ3	E2, E3	ND	2 d for E2	0.5	Ke et al., 2007 [39]
*Aminobacter* sp. KC6	E1, E2	ND	7 d for E2	3	Yu et al., 2007 [3]
*Acinetobacter* sp. BP8, BP10	E1, E2, E3, EE2	ND	1 d for E1, E3	3.2, 3.6 for E1, E3	Pauwels et al., 2008 [23]
		ND	2 d for E2	2.3 for others	
*Acidovorax* sp.	NP	80%–90%	5 d	50–100	Watanabe et al., 2012 [24]
*Bacillus* sp. E2Y1, E2Y4	E1, E2	ND	4 d for E2Y4	1 for E2	Jiang et al., 2010 [34]
			6 d for E2T1	1for E2	
*Novosphingobium* sp. JEM-1	E1, E2, EE2	81%	9 h for E2	1.3	Hashimoto et al., 2010 [25]
*Pseudomonas aeruginosa* BP3, BP7	E1, E2, E3, EE2	ND	1 d for E1, E3	3.2, 3.6 for E1, E3	Pauwels et al., 2008 [23]
		ND	2 d for E2	2.3 for others	
*Pseudomonas* sp*.*	NP	20%–25%	5 d	50–100	Watanabe et al., 2012 [24]
*Pseudomonas* sp*.*	DES	80%	1 d	10	Zhang et al., 2013 [26]
*Ralstonia pickettii* BP2	E1, E2, E3, EE2	ND	1 d	3.2, 3.6 for E1,E3	Pauwels et al., 2008 [23]
		ND	2 d	2.3 for others	
*Rhodococcus* sp. ED7,	E1, E2	90%	120 h	200 for E1, E2	Kurisu et al., 2010 [27]
*Rhodococcus zopfii* ATCC 51349, ATCC 13557	EE2	47%	13 h	1.4	O’Grady et al., 2009 [40]
*Stenotrophomonas maltophilia* ZL1	E2	100%	100 h	3.3	Li et al., 2012 [41]
*Sphingobacterium* sp. JCR5	EE2	87%	10 d	30	Ren et al., 2007 [42]
*Sphingomonas* sp. KC8	E1, E2	ND	7 d for E2	3	Yu et al., 2007 [3]

ND: the concentration of estrogen was undetectable. E1: estrone. E2: 17β-estradiol. E3: estriol. EE2: ethinyloestradiol. DES: diethylstilbestrol. NP: Nonylphenol.
